# Identification of biomarkers of chromophobe renal cell carcinoma by weighted gene co-expression network analysis

**DOI:** 10.1186/s12935-018-0703-z

**Published:** 2018-12-17

**Authors:** Xiaomao Yin, Jianfeng Wang, Jin Zhang

**Affiliations:** Department of Urology, Renji Hospital, Shanghai Jiao Tong University School of Medicine, 1630 Dong Fang Road, Shanghai, 200127 China

**Keywords:** Chromophobe renal cell carcinoma, Weighted gene co-expression network analysis (WGCNA), Biomarker

## Abstract

**Background:**

Chromophobe renal cell carcinoma (ChRCC) is the second common subtype of non-clear cell renal cell carcinoma (nccRCC), which accounting for 4–5% of renal cell carcinoma (RCC). However, there is no effective bio-marker to predict clinical outcomes of this malignant disease. Bioinformatic methods may provide a feasible potential to solve this problem.

**Methods:**

In this study, differentially expressed genes (DEGs) of ChRCC samples on The Cancer Genome Atlas database were filtered out to construct co-expression modules by weighted gene co-expression network analysis and the key module were identified by calculating module-trait correlations. Functional analysis was performed on the key module and candidate hub genes were screened out by co-expression and MCODE analysis. Afterwards, real hub genes were filter out in an independent dataset GSE15641 and validated by survival analysis.

**Results:**

Overall 2215 DEGs were screened out to construct eight co-expression modules. Brown module was identified as the key module for the highest correlations with pathologic stage, neoplasm status and survival status. 29 candidate hub genes were identified. GO and KEGG analysis demonstrated most candidate genes were enriched in mitotic cell cycle. Three real hub genes (SKA1, ERCC6L, GTSE-1) were selected out after mapping candidate genes to GSE15641 and two of them (SKA1, ERCC6L) were significantly related to overall survivals of ChRCC patients.

**Conclusions:**

In summary, our findings identified molecular markers correlated with progression and prognosis of ChRCC, which might provide new implications for improving risk evaluation, therapeutic intervention, and prognosis prediction in ChRCC patients.

**Electronic supplementary material:**

The online version of this article (10.1186/s12935-018-0703-z) contains supplementary material, which is available to authorized users.

## Background

Renal cell carcinoma (RCC) is a heterogenous disease, which is composed of ccRCC and nccRCC [1]. Over the past few years, targeted therapies have significantly improved overall survival (OS) and relapse free survival (RFS) of patients with ccRCC [2]. However, ascribing to relatively low incidence (25–30%) and rare clinical trails of nccRCC, the optimal targeted therapies for nccRCC patients still remain uncertain [3]. ChRCC, taking up 4–5% of RCC, is the second common subtype of nccRCC. Although the tumor grade or stage of ChRCC is relatively low, there is no significant difference between patients with localized ChRCC and ccRCC in 5-year cancer-specific survival rates (P = 0.98) [4]. Due to the poor outcomes of ChRCC, it’s urgent to identify novel molecular biomarkers to evaluate the prognosis of ChRCC patients, which might help to assess the malignancy and provide therapeutic potential for this disease.

WGCNA is a method commonly used to explore the complex relationships between genes and phenotypes. This method is able to transform gene expression data into co-expression modules and provide insights into signaling networks that may be responsible for phenotypic traits of interests [5–7]. WGCNA is widely used in various biological processes, such as cancer, neuroscience and genetic data analysis, which is quite helpful for the identification of potential biomarkers or therapeutic targets [8–11]. Not only can it analyses mRNA level of tumor samples, but also work on microRNA or lncRNA datasets of neoplasms to find candidate biomarkers for prognosis and treatment [12, 13].

In this study, WGCNA method was firstly used to analyze clinic traits and gene expression data of ChRCC samples provided by TCGA database to identify key genes associated with tumor prognosis and progression. Our findings may be very beneficial to assess malignant potential of ChRCC and offer therapeutic methods to this neoplasm.

## Methods

### Data sources and data preprocessing

Gene expression data and patient clinic traits of ChRCC were downloaded from The Cancer Genome Atlas (TCGA) database (https://cancergenome.nih.gov/). Annotation information of microarray was used to match probes with corresponding genes. The average value was calculated out for those genes corresponding to more than one probes, while probes matched with more than one gene were eliminated.

### Screening for differentially expressed genes

The “DEseq2” R package was used to screen DEGs between ChRCC samples and paired normal tissues in the expression data. The DEGs threshold was set at a |log2FoldChange| > 0.585 and adj.P.value < 0.05. After DEGs were screen out, flashClust tool package in R language was used to perform cluster analysis of ChRCC samples.

### Co-expression network construction and module analysis

Firstly, expression data profile of DEGs was tested by to check if they were the good samples and good genes. Afterwards, power value was screened out by WGCNA [14] algorithm which is implemented in the R software package (http://www.r-project.org/). Scale independence and average connectivity degree of modules with different power value was tested by gradient method (the power value ranging from 1 to 20). The appropriate power value was determined when the degree of independence was above 0.85 and average connectivity degree is relatively higher [8]. Once the power value was determined, the scale-free gene co-expression networks were constructed by WGCNA algorithm. Besides, the corresponding genes information of each module was extracted out. The minimum number of genes in each module was set as 50 for the high reliability of the results.

### Interaction analysis of co-expression modules of ChRCC

After co-expression modules were identified by WGCNA algorithm. Heatmap was painted to describe the strength of the interactions between different modules by heatmap function embedded in R language.

### Construct module-trait relationships of ChRCC and key module identification

The correlation between module eigengenes (MEs) and phenotype (clinic traits) was used to evaluated module-trait associations. MEs were considered as the major component in the principal component analysis for each gene module. We calculated the correlation between MEs and clinical trait to identify the relevant module. Gene significance (GS), which was defined as the log10 transformation of the P value (GS = lgP) in the linear regression between gene expression and clinical information, was calculated to evaluate correlation strength. Modules with the highest correlation coefficients among all modules were usually considered as the key module and selected for further analysis.

### Functional enrichment analysis of the key module

The information of genes in key modules was upload to Enrichr online database to perform GO and KEGG pathway analysis [15, 16]. Analysis results were extracted out under the condition of P < 0.05 after correction [17]. The top 10 GO terms were visualized if there were more than ten terms, so as to KEGG pathways.

### Selection of candidate hub genes

Hub genes, with highly intramodular connectivity, have been shown to be functionally significant. In this study, candidate hub genes were defined by module connectivity, measured by module membership (MM) > 0.8 and clinical trait relationship, measured by significance of the Pearson’s correlation (GS. Pathologic stage > 0.2) [18]. Furthermore, we uploaded all genes in the hub module to the STRING database, choosing confidence > 0.4 to construct protein–protein interaction (PPI). After setting degree cut-off = 5, node score cut-off = 0.2, k-core = 2, and max. depth = 100, the most significant sub-module was selected by using plug-in MCODE [9]. Genes both in co-expression network and MCODE sub-module were regarded as candidate hub genes for further analysis.

### Hub genes identification and validation

Another independent dataset GSE15641 containing six ChRCC samples and 23 normal tissues was extracted and performed on GEO2R analysis (https://www.ncbi.nlm.nih.gov/geo/). |log2FoldChange| > 1 and P.Value < 0.05 as criterion, DEGs were identified. Candidate hub genes were mapped to DEGs in GSE15641 to find the real hub genes. For further validation, real hub genes were upload to online TCGA database to perform survival analysis (http://gepia.cancer-pku.cn/). ChRCC patients were divided into two groups according to median expression of each hub gene (high vs. low) and Kaplan–Meier survival analysis was conducted.

### Statistical analyses

Two-tailed Student’s t-test was used for significance of differences between groups. Statistical analyses were performed with GraphPad Prism 6.02. Statistical significance was set at probability values of P < 0.05.

## Results

### DEGs screening

The gene expression profile of 66 ChRCC samples were download from TCGA database. A total of 2215 DEGs (1741 up-regulated and 384 down-regulated) between ChRCC samples and normal tissues were screened out under the threshold of |log2FoldChange| > 0.585 and adj.P.Value < 0.05. These 2215 DEGs were then selected for subsequent analysis.

### Construction of co-expression modules of ChRCC

**Fig. 1:**
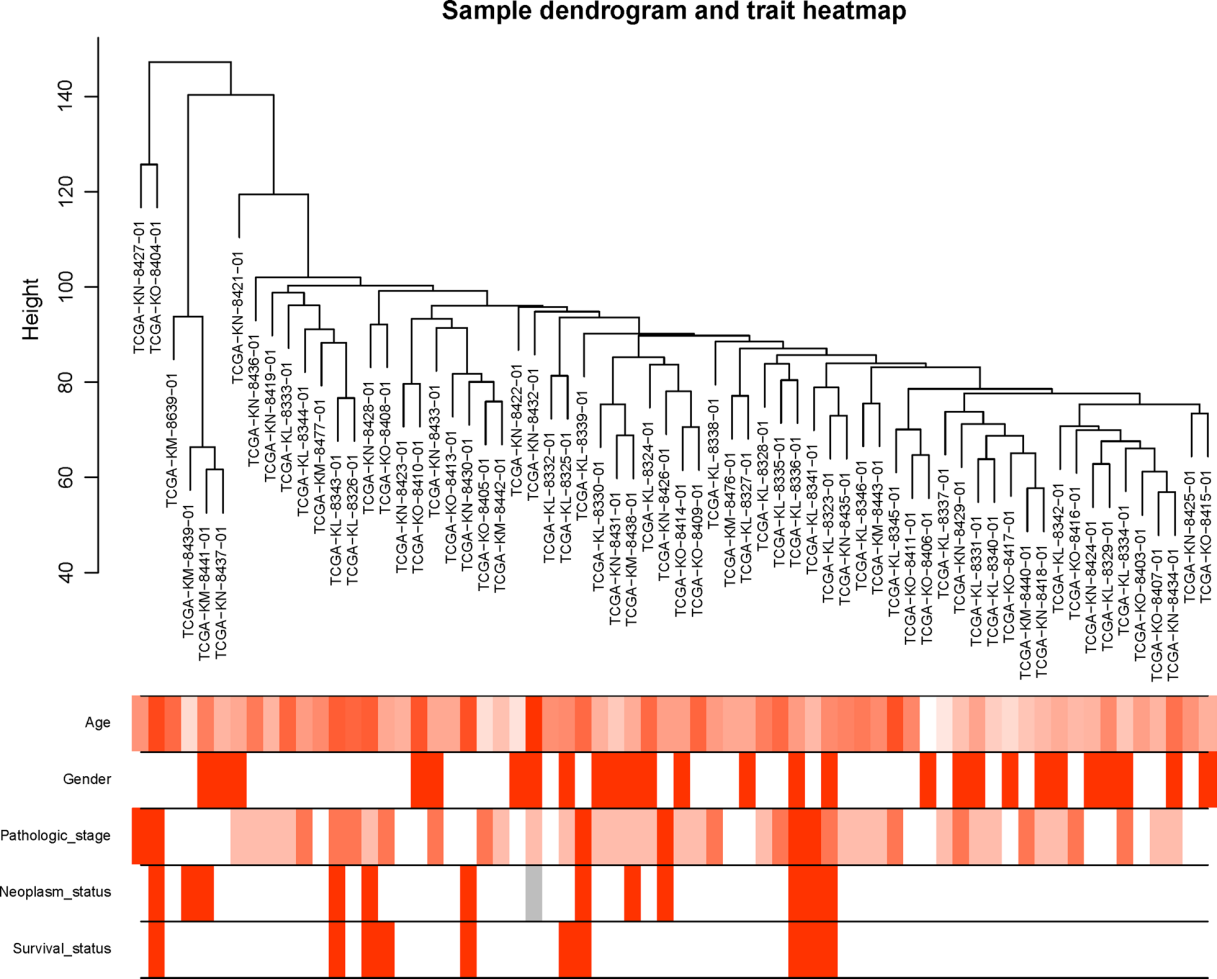
Clustering dendrogram of 66 tumor samples and heatmaps of clinical traits. The clustering was based on the expression data of DEGs between ChRCC samples and non-tumor samples. The color intensity in heatmaps was proportional to older age, higher pathological stage. In neoplasm and survival status, white means patient neoplasm free or live, while red means patient with tumor or dead

**Fig. 2:**
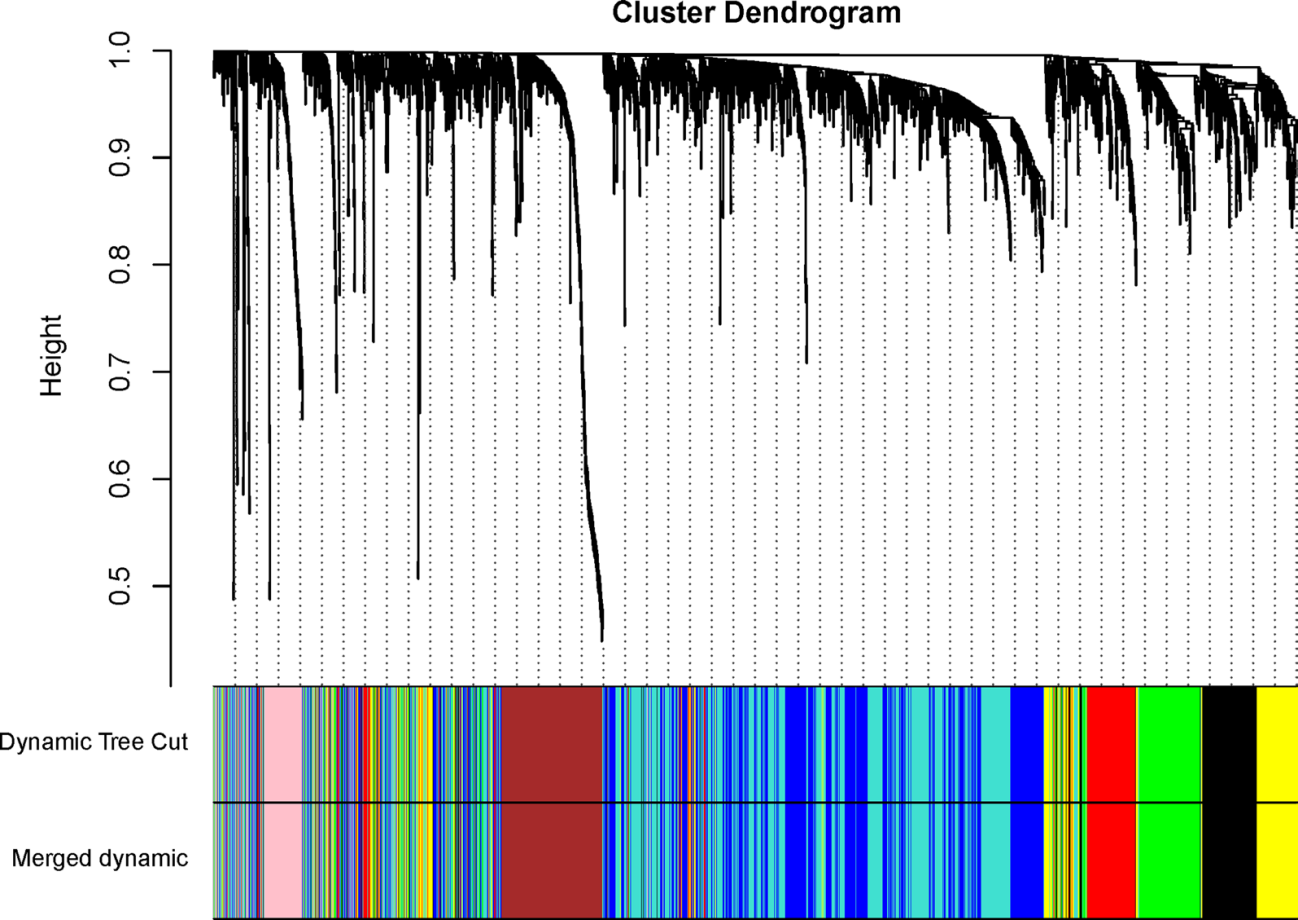
Clustering dendrogram of DEGs, with dissimilarity based on topological overlap, together with assigned module colors

**Fig. 3:**
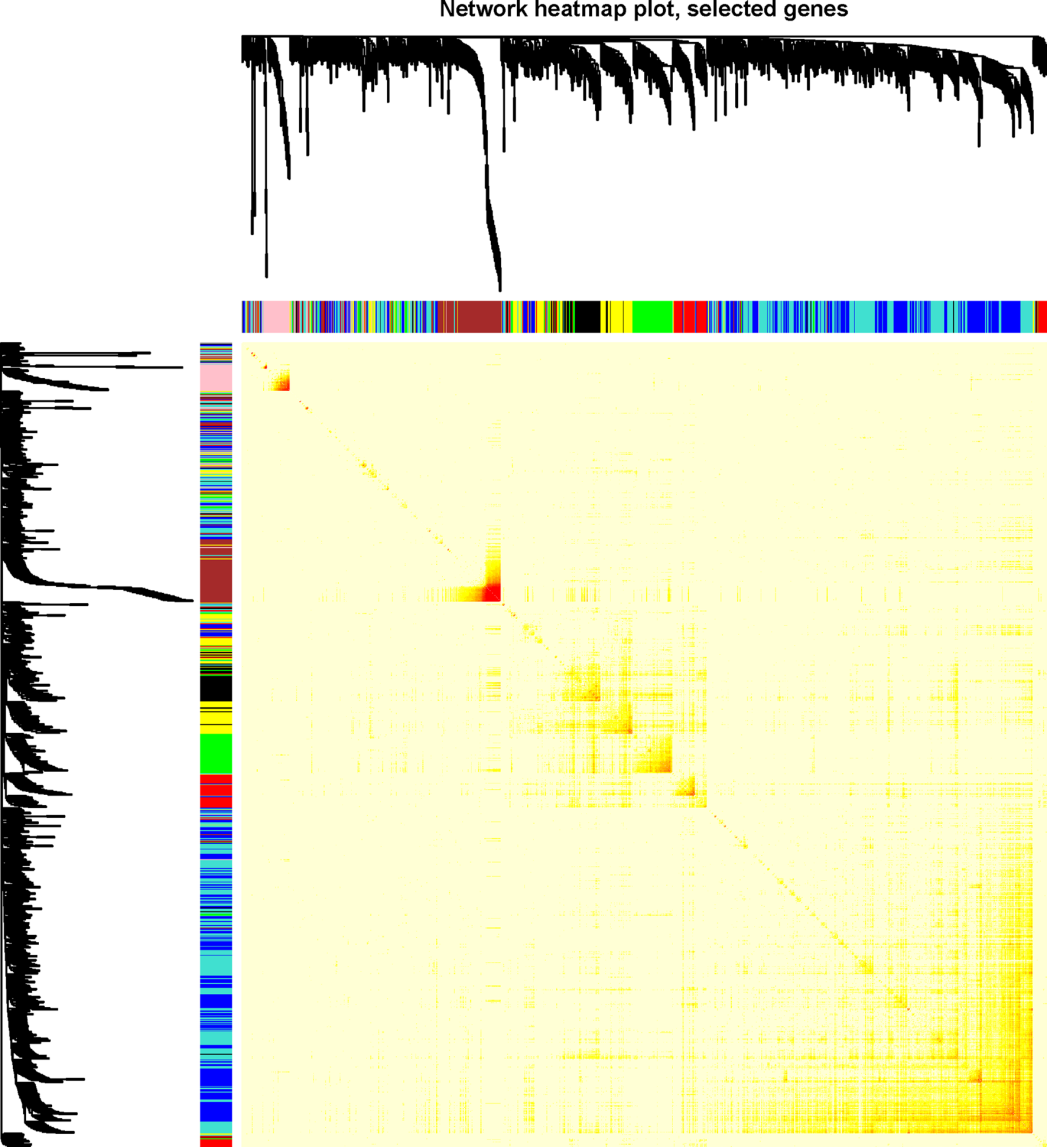
Visualizing the gene network using a heatmap plot. The heatmap depicts the Topological Overlap Matrix (TOM) among all genes in the analysis. Light color represents low overlap and progressively darker red color represents higher overlap. The gene dendrogram and module assignment are also shown along the left side and the top

All DEGs were included for constructing co-expression modules by WGCNA algorithm. The “flashClust tool” package was used to perform the cluster and trait analysis and results were shown in (Fig. 1). First of all, the appropriate power value was screened out (Additional file 1: Fig S1). When the power value was equal to 5, scale free networks were constructed with independence degree up to 0.85 and the relatively higher average connectivity (mean connectivity = 6.04). As a result, eight distinct gene co-expression modules were identified by the appropriate power value (5) in ChRCC after excluding the grey module. Constructed modules painted with different colors and cluster trees of DEGs were shown in (Fig. 2). Gene numbers and module names were shown in Table 1. Interactions between eight co-expression modules were subsequently analyzed. The heatmap demonstrates the Topological Overlap Matrix (TOM) among all genes in the analysis. Light color represents low overlap and darker red color represents higher overlap. As a result, each module showed independent validation to each other (Fig. 3).

**Table 1:** The number of genes in eight constructed modules

Modules	Freq
Black	148
Blue	498
Brown	222
Green	179
Pink	82
Red	149
Turquoise	574
Yellow	199

### Identification of key modules corresponding to clinic traits

**Fig. 4:**
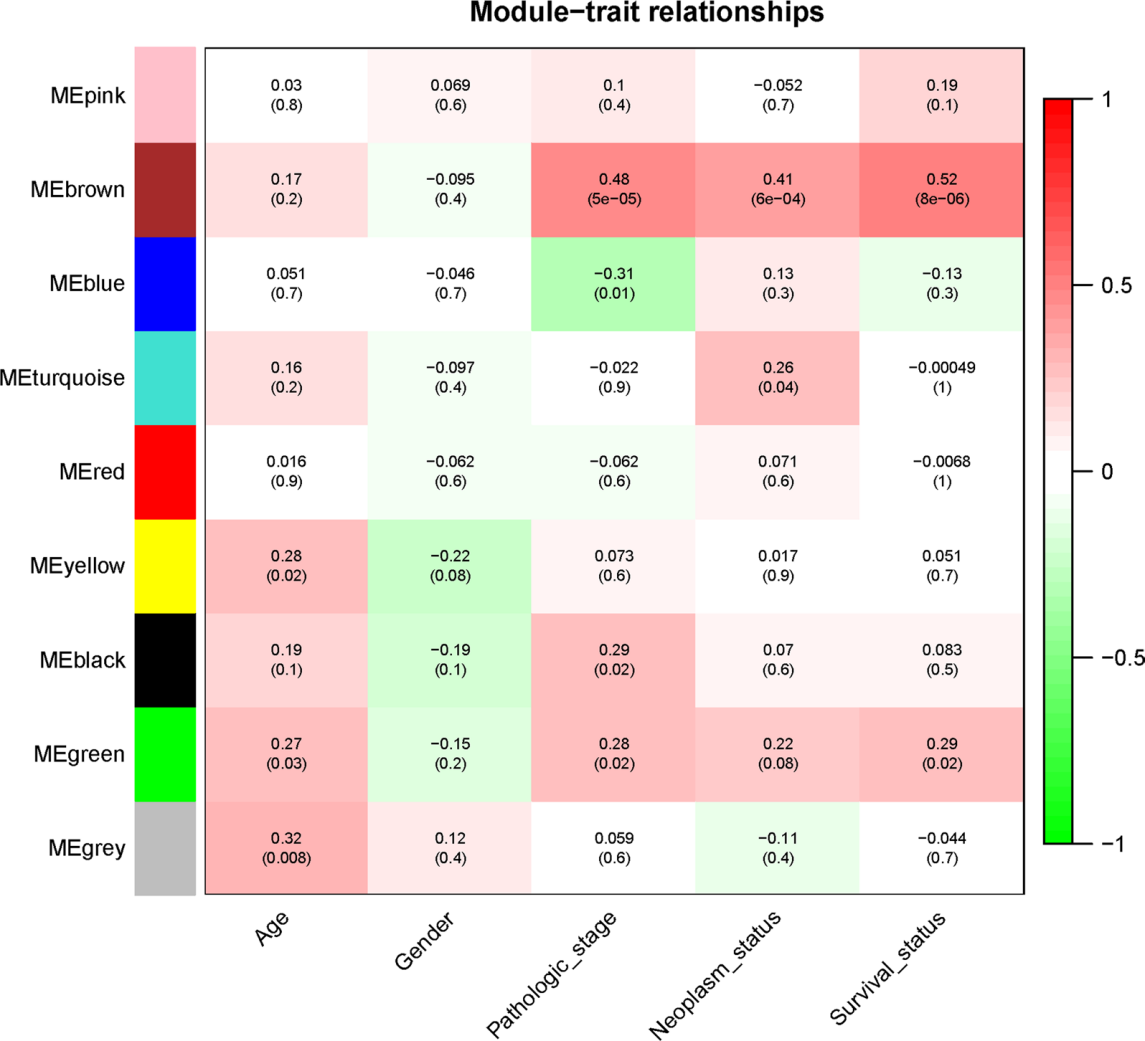
Module-trait heatmap. Each row corresponds to a module eigengene, column to a trait. Each cell contains the corresponding correlation and P-value

The correlations between module eigengene and clinic traits were shown in (Fig. 4). The brown module was selected as key module for taking up top three highest correlations with progression and survival of ChRCC (R^2^ = 0.48 and P = 5e^−05^ with pathologic stage, R^2^ = 0.41 and P = 6e^−04^ with neoplasm status, R^2^ = 0.52 and P = 8e^−06^ with living status). Afterwards, we plotted scatter plots of GS vs. MM (module membership) in the brown modules with clinic traits respectively (Additional file 2: Fig S2).

### Functional enrichment analysis of genes in the key module

**Fig. 5:**
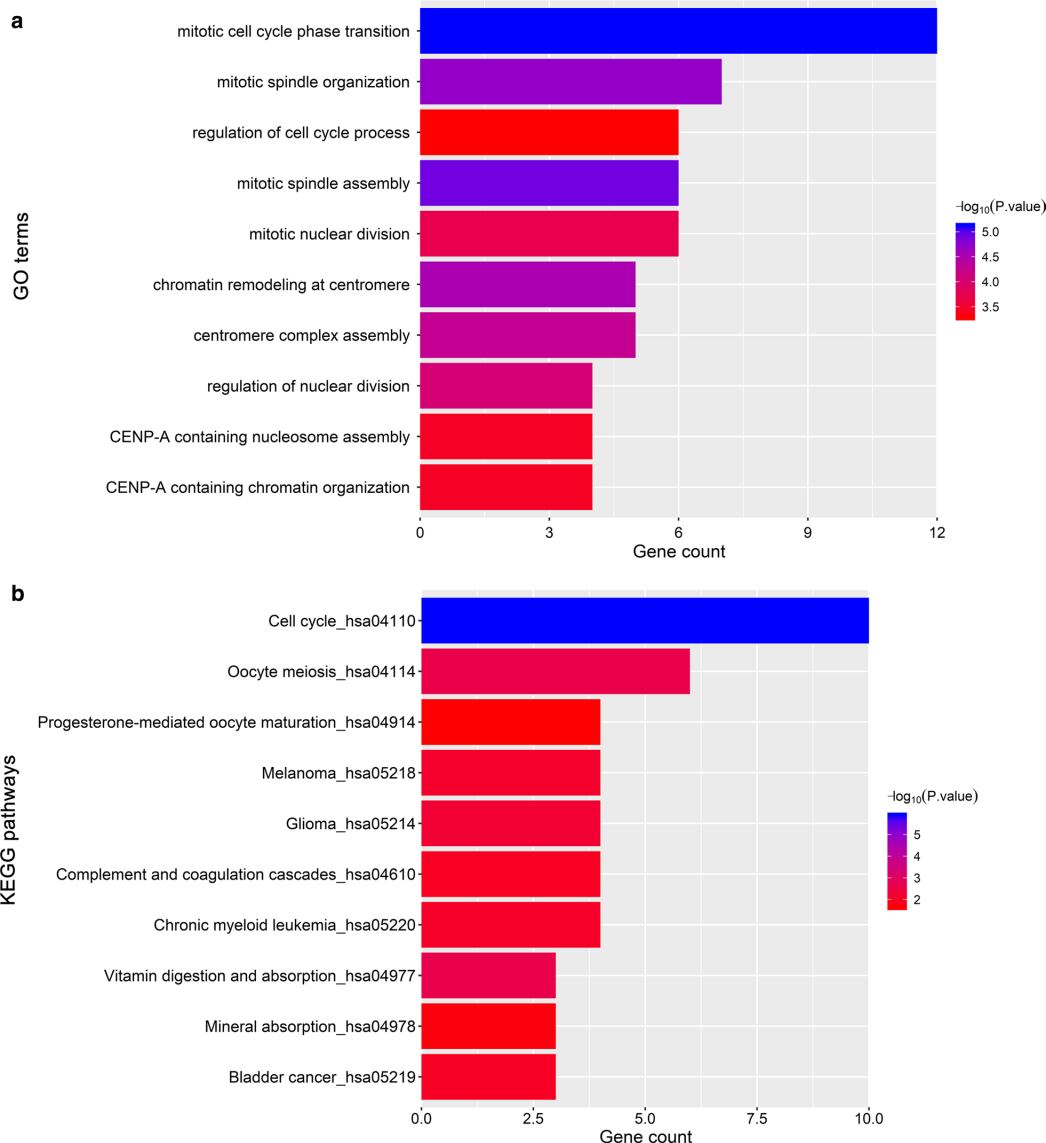
Functional analysis. **a** Gene ontology analysis of genes in brown module. **b** KEGG pathway enrichment analysis of genes in brown module. The x-axis shows the − log10 (*P*-value) of each term and the y-axis shows the GO and KEGG pathway terms

To obtain further insight into the function of genes in the hub module, all genes in the brown module were uploaded to Enrichr online database to conduct GO and KEGG pathway analysis. According to P-value of each term, top 10 biological process and KEGG pathways were extracted out (Additional file 3: Table S1 and Additional file 4: Table S2) and visualized. GO analysis results showed genes in brown module significantly enriched in mitotic cell cycle transition, mitotic spindle assembly, mitotic spindle organization, regulation of cell cycle process, etc. KEGG pathway analysis revealed cell cycle, oocyte meiosis, progesterone-mediated oocyte maturation pathways were significantly enriched (Fig. 5). All these results implied that dysfunctional mitotic cell cycle may contribute to tumorigeneses of ChRCC.

### Selection of candidate hub genes

**Fig. 6:**
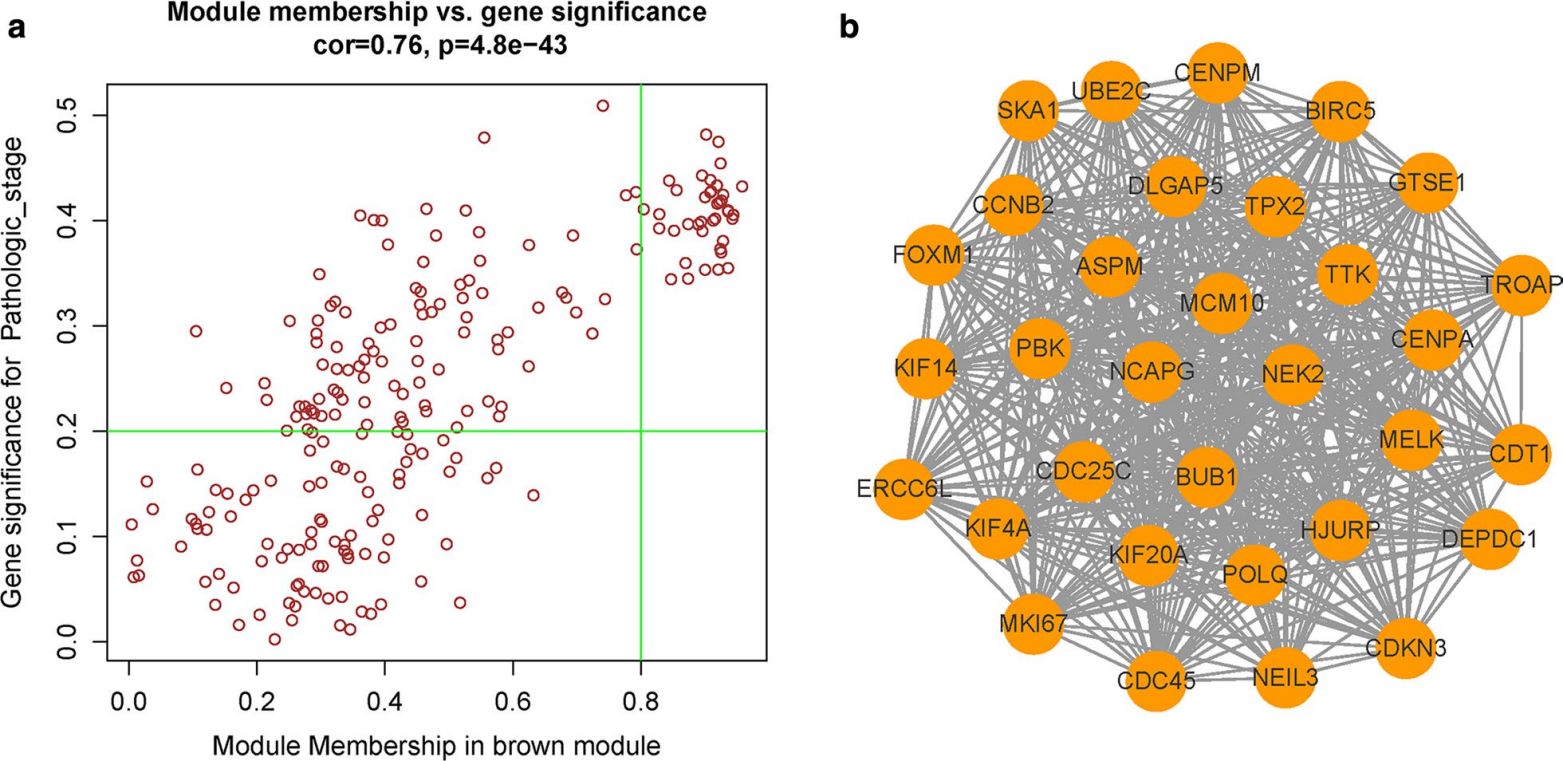
Candidate hub gene identification. **a** Scatter plot of module eigengenes in brown module. The vertical line represents cutoff of module membership = 0.8, the horizontal line represents cutoff of gene significances in pathologic stage = 0.2. Genes on upper right were selected out. **b** PPI network of genes in MCODE sub-module

Under the condition of MM > 0.8 and GS. Pathologic stage > 0.2, 39 genes in brown module were taken out (Fig. 6a). PPI network were constructed under the cutoff of confidence > 0.4, 32 genes were filtered out after MCODE process (Fig. 6b). 29 candidate genes identified both in co-expression network and MCODE sub-module were show in Table 2.

**Table 2:** Candidate hub genes identified by co-expression and MCODE methods

Gene symbol	Co-expression analysis				MCODE analysis	
	GS.Pathologic_stage	GS.Neoplasm_status	GS.Survival_status	MM	Connectivity degree	MCODE_score
ASPM	0.3698	0.3874	0.5063	0.9254	50	39
KIF14	0.4161	0.3991	0.4492	0.9193	50	39
TPX2	0.4019	0.3765	0.4620	0.9429	53	39
NEK2	0.3730	0.3988	0.5093	0.9240	52	39
MKI67	0.4088	0.4114	0.4970	0.9361	53	39
NCAPG	0.3806	0.4425	0.5053	0.9282	51	39
DLGAP5	0.3549	0.4440	0.5035	0.9360	54	39
KIF4A	0.4325	0.4104	0.4824	0.9581	56	39
KIF20A	0.4190	0.4013	0.4783	0.9259	50	39
FOXM1	0.4174	0.4091	0.4626	0.9225	51	39
CDKN3	0.3534	0.4401	0.5048	0.9209	49	39
BIRC5	0.3988	0.4194	0.4129	0.8940	48	39
CDC25C	0.4268	0.4004	0.4570	0.9084	47	39
CCNB2	0.3533	0.3397	0.4443	0.9002	46	39
PBK	0.3964	0.4196	0.4888	0.8895	47	39
POLQ	0.4818	0.3971	0.5722	0.9018	45	39
MELK	0.4246	0.3989	0.4592	0.9284	51	39
CENPA	0.4223	0.4006	0.4285	0.9002	47	39
SKA1	0.4019	0.4533	0.5004	0.9159	51	39
HJURP	0.4001	0.3558	0.4416	0.9128	50	39
TROAP	0.3900	0.3643	0.4276	0.8959	46	39
BUB1	0.3927	0.3514	0.4558	0.8284	39	39
ERCC6L	0.4544	0.3660	0.5429	0.9243	50	39
TTK	0.4380	0.3833	0.5951	0.8440	40	39
MCM10	0.4108	0.4499	0.3941	0.8043	41	39
CDC45	0.3597	0.4241	0.4214	0.8697	45	39
GTSE1	0.4334	0.2835	0.4238	0.9186	50	39
NEIL3	0.4386	0.4524	0.4932	0.9085	48	39
DEPDC1	0.4054	0.4806	0.5418	0.9443	54	39

### Identification and validation of hub genes

**Fig. 7:**
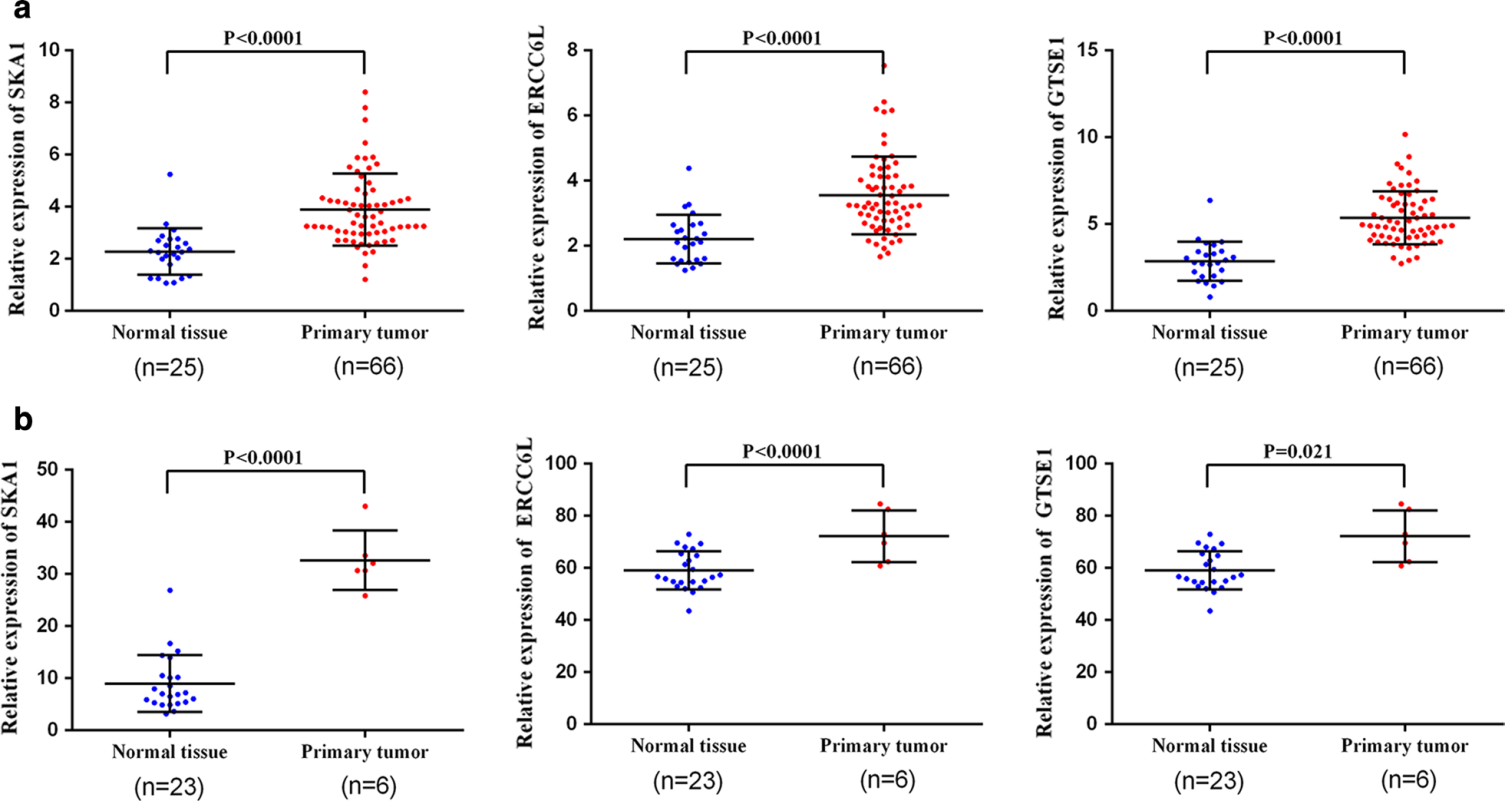
Expression levels of real hub genes. **a** Expression levels of real hub genes between tumor samples and normal tissues in TCGA database. **b** Expression levels of real hub genes between tumor samples and normal tissues in GSE15641 dataset

**Fig. 8:**
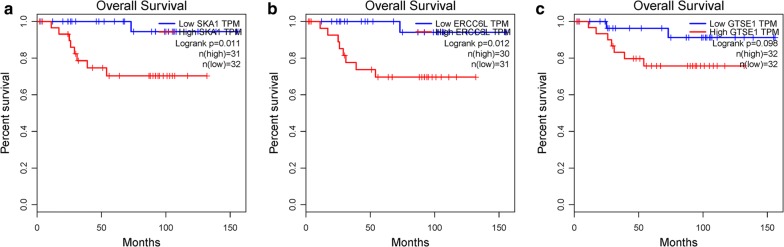
Validation of real hub genes by survival analysis between high expression group (red line) and low expression group (blue line). **a** SKA1, **b** ERCC6L, **c** GTSE1

1794 DEGs (884 up-regulated, 910 down-regulate) were identified in GSE15641. All candidate genes were mapped to DEGs in GSE15641, four genes (SKA1, ERCC6L, POLQ, GTSE1) were found to be differentially expressed both in TCGA and GSE15641 datasets. While expressions of POLQ in two datasets were opposite (up-regulated in TCGA, down-regulated in GSE15641). Thus, SKA1, ERCC6L and GTSE1 were regarded as the real hub genes in ChRCC by WGCNA analysis. Expressions of real hub genes between ChRCC samples and normal tissues were shown in (Fig. 7). Survival analysis of these genes showed over-expressions of SKA1 and ERCC6L are significantly related to shorter overall survival time (Fig. 8).

## Discussion

ChRCC is an uncommon, but not rare, malignant disease representing ~ 5% of histologic spectrum of cancer arising from kidney [19]. However, patients with ChRCC have a relatively low risk of tumor progression and cancer-specific death [20], the clinical outcomes of target therapies between ChRCC and ccRCC were not significantly different in metastatic disease [21]. To the best of our knowledge, there are no effective treatments for patients with metastatic ChRCC or any preventions for tumor recurrence. Thus, it’s important to get a better understanding of molecular mechanism of ChRCC and identify potential biomarkers to evaluate the biological behavior of this malignancy.

WGCNA has many prominent advantages over other methods since the analysis explores associations between co-expression modules and clinic traits and the results had much higher reliability and biological significance [22]. In this study, a total of eight co-expression modules were constructed by the 2215 DEGs from the 66 human ChRCC samples by WGCNA method, which was used to detect the relationship between ChRCC transcriptome and clinic traits. We calculated the correlations between co-expression modules and pathologic stage, neoplasm status and living status. Brown module with the highest correlation with clinic traits were regarded as the key module to explore primary cause for disease progression.

For further analysis, genes in brown module with high module membership (> 0.8) and significance of correlations with pathologic stage (> 0.2) were filtered out. Meanwhile, MCODE analysis was performed on brown module to find a panel of genes with high connective degrees [9]. 29 candidate hub genes were identified by overlapping results of co-expression analysis and MCODE analysis. GO and KEGG analysis showed that dysregulation of cell cycle may be the underlying mechanism of tumorigeneses of ChRCC.

29 candidate hub genes were subsequently validated in GSE15641 dataset, four candidate genes (SKA1, ERCC6L, POLQ, GTSE1) differentially expressed both in TCGA and GSE15641 database were considered as real hub genes. After excluding POLQ for conflicting expression level in two databases, two of three hub genes (SKA1, ERCC6L) were found to be significantly correlated with shorter overall survival time under their over expressions.

SKA1 was a microtubule-binding subcomplex of the outer kinetochore which is essential for proper chromosome segregation. It played an important role on tumorigenesis in multiple malignancies [23, 24]. SKA1 over-expression led to cancerization in human prostate epithelial cells via the induction of centriole over-duplication [25]. Depletion of SKA1 inhibited cell proliferation in gastric cancer by blocking cell cycle in S phase [26]. What’s more, SKA1 was proved to be an oncogene in ccRCC which could be down-regulated by antitumor miR-10a-5p transfection [27]. ERCC6L gene, also named PLK1-interacting checkpoint helicase (PICH), was a member of the SNF2 protein family (SWI/SNF catalytic subunit SNF2). It was a mitotic target and substrate of polo-like kinases (PLKs) which regulates multiple processes in mammalian cell mitosis [28]. Downregulation of ERCC6L decreased cell viability in RCC cell lines by blocking mitogen-activated protein kinase (MAPK) signaling pathway and interactions with protein PLK1 [29]. Protein encoded by GTSE-1 modulated cell migrations in an EB1-dependent manner. Up-regulation of GTSE1 expression could be associated with increased invasive potential in breast cancer [30]. Depletion of GTSE-1 enhanced mitotic centromere-associated kinesin (MCAK) activity in mitotic cells, leading to chromosomal instability (CIN) which was presented in most solid tumors [31]. Silencing GTSE-1 expression inhibits proliferation and invasion of hepatocellular carcinoma cells [32]. Based on many studies about three hub genes, we could find their important roles in tumorigenesis and metastasis. Targeting these biomarkers and other candidate hub genes participating in cell cycles could provide therapeutic potentials for ChRCC.

Some limitations exiting in our studies should be mentioned. The most vital genes out of 29 candidate hub genes couldn’t been filtered out under restrictions of the bioinformatics methods. Multi-central and large sample studies on ChRCC expression profiles were needed for validation of our findings. However, this couldn’t be fulfilled due to limited online databases for ChRCC.

## Conclusions

Our study used weighted gene co-expression analysis to construct a gene co-expression network, identify and validate the key module and hub genes associated with the progression and prognosis of ChRCC. Functional analysis of hub genes reveal mitotic cell cycle dysregulation could be the underlying mechanism of ChRCC. Three real hub genes including SKA1, ERCC6L and GTSE-1 were identified. SKA1 and ERCC6L were validated in association with poor prognosis of ChRCC. However, further molecular biological experiments are needed to confirm the function of these biomarkers in ChRCC.

## Additional files

**Additional file 1: Fig S1.** Analysis of network topology for various soft thresholding powers. The left panel shows the scale-free fit index (y-axis) as a function of the soft-thresholding power (x-axis). The right panel displays the mean connectivity (degree, y-axis) as a function of the soft-thresholding power (x-axis).

**Additional file 2: Fig S2.** (a) Scatterplot of Gene Significance (GS) for pathologic stage vs. Module Membership (MM) in the brown module. (b) scatterplot of Gene Significance (GS) for neoplasm status vs. Module Membership (MM) in the brown module. (c) scatterplot of Gene Significance (GS) for survival status vs. Module Membership (MM) in the brown module.

**Additional file 3: Table S1.** GO enrichment analysis in brown module.

**Additional file 4: Table S2.** KEGG pathways enrichment in brown module.

## Abbreviations

ChRCC: chromophobe renal cell carcinoma
nccRCC: non-clear cell renal cell carcinoma
RCC: renal cell carcinoma
TCGA: The Cancer Genome Atlas
WGCNA: weighted gene co-expression network analysis
GO analysis: gene ontology analysis
ccRCC: clear cell renal cell carcinoma
OS: overall survival
RFS: relapse free survival
GS: gene significance
MM: module membership

## References

[1] Barata PC, Rini BI (2017). Treatment of renal cell carcinoma: current status and future directions. CA Cancer J Clin.

[2] Jonasch E, Gao JJ, Rathmell WK (2014). Renal cell carcinoma. BMJ.

[3] Bellmunt J, Dutcher J (2013). Targeted therapies and the treatment of non-clear cell renal cell carcinoma. Ann Oncol.

[4] Lee WK, Byun SS, Kim HH, Rha KH, Hwang TK, Sung GT, Lee W, Lim JS, Jeong YB, Kwon TG (2010). Characteristics and prognosis of chromophobe non-metastatic renal cell carcinoma: a multicenter study. Int J Urol.

[5] Horvath S, Zhang B, Carlson M, Lu KV, Zhu S, Felciano RM, Laurance MF, Zhao W, Qi S, Chen Z, Lee Y, Scheck AC, Liau LM, Wu H, Geschwind DH, Febbo PG, Kornblum HI, Cloughesy TF, Nelson SF, Mischel PS (2006). Analysis of oncogenic signaling networks in glioblastoma identifies ASPM as a molecular target. Proc Natl Acad Sci USA.

[6] Mao Q, Zhang L, Zhang Y, Dong G, Yang Y, Xia W, Chen B, Ma W, Hu J, Jiang F, Xu L (2018). A network-based signature to predict the survival of non-smoking lung adenocarcinoma. Cancer Manag Res.

[7] Yuan L, Qian G, Chen L, Wu CL, Dan HC, Xiao Y, Wang X (2018). Co-expression network analysis of biomarkers for adrenocortical carcinoma. Front Genet.

[8] Chen L, Yuan L, Wang Y, Wang G, Zhu Y, Cao R, Qian G, Xie C, Liu X, Xiao Y, Wang X (2017). Co-expression network analysis identified FCER1G in association with progression and prognosis in human clear cell renal cell carcinoma. Int J Biol Sci.

[9] Yuan LS, Zeng G, Chen L, Wang G, Wang XL, Cao XY, Lu MX, Liu XF, Qian GF, Xiao Y, Wang XH (2018). Identification of key genes and pathways in human clear cell renal cell carcinoma (ccRCC) by co-expression analysis. Int J Biol Sci.

[10] Spiers H, Hannon E, Schalkwyk LC, Smith R, Wong CCY, O’Donovan MC, Bray NJ, Mill J (2015). Methylomic trajectories across human fetal brain development. Genome Res.

[11] Liu Q, Jiang C, Xu J, Zhao MT, Van Bortle K, Cheng X, Wang GW, Chang HY, Wu JC, Snyder MP (2017). Genome-wide temporal profiling of transcriptome and open chromatin of early cardiomyocyte differentiation derived from hiPSCs and hESCs. Circ Res.

[12] Giulietti M, Occhipinti G, Principato G, Piva F (2017). Identification of candidate miRNA biomarkers for pancreatic ductal adenocarcinoma by weighted gene co-expression network analysis. Cell Oncol.

[13] Giulietti M, Righetti A, Principato G, Piva F (2018). LncRNA co-expression network analysis reveals novel biomarkers for pancreatic cancer. Carcinogenesis.

[14] Langfelder P, Horvath S (2008). WGCNA: an R package for weighted correlation network analysis. BMC Bioinform.

[15] Kuleshov MV, Jones MR, Rouillard AD, Fernandez NF, Duan Q, Wang Z, Koplev S, Jenkins SL, Jagodnik KM, Lachmann A, McDermott MG, Monteiro CD, Gundersen GW, Ma’ayan A (2016). Enrichr: a comprehensive gene set enrichment analysis web server 2016 update. Nucleic Acids Res.

[16] Chen EY, Tan CM, Kou Y, Duan Q, Wang Z, Meirelles GV, Clark NR, Ma’ayan A (2013). Enrichr: interactive and collaborative HTML5 gene list enrichment analysis tool. BMC Bioinform.

[17] Ashburner M, Ball CA, Blake JA, Botstein D, Butler H, Cherry JM, Davis AP, Dolinski K, Dwight SS, Eppig JT, Harris MA, Hill DP, Issel-Tarver L, Kasarskis A, Lewis S, Matese JC, Richardson JE, Ringwald M, Rubin GM, Sherlock G (2000). Gene ontology: tool for the unification of biology. The Gene Ontology Consortium. Nat Genet.

[18] Chen L, Yuan L, Qian K, Qian G, Zhu Y, Wu CL, Dan HC, Xiao Y, Wang X (2018). Identification of biomarkers associated with pathological stage and prognosis of clear cell renal cell carcinoma by co-expression network analysis. Front Physiol.

[19] Reuter VE (2006). The pathology of renal epithelial neoplasms. Semin Oncol.

[20] Volpe A, Novara G, Antonelli A, Bertini R, Billia M, Carmignani G, Cunico SC, Longo N, Martignoni G, Minervini A, Mirone V, Simonato A, Terrone C, Zattoni F, Ficarra V, LUN-PF A (2012). Chromophobe renal cell carcinoma (RCC): oncological outcomes and prognostic factors in a large multicentre series. BJU Int.

[21] Keizman D, Sarid D, Lee JL, Sella A, Gottfried M, Hammers H, Eisenberger MA, Carducci MA, Sinibaldi V, Neiman V, Rosenbaum E, Peer A, Neumann A, Mermershtain W, Rouvinov K, Berger R, Yildiz I, Grp TO (2016). Outcome of patients with metastatic chromophobe renal cell carcinoma treated with sunitinib. Oncologist.

[22] Chou WC, Cheng AL, Brotto M, Chuang CY (2014). Visual gene-network analysis reveals the cancer gene co-expression in human endometrial cancer. BMC Genom.

[23] Tian F, Xing X, Xu F, Cheng W, Zhang Z, Gao J, Ge J, Xie H (2015). Downregulation of SKA1 Gene expression inhibits cell growth in human bladder cancer. Cancer Biother Radiopharm.

[24] Qin X, Yuan B, Xu X, Huang H, Liu Y (2013). Effects of short interfering RNA-mediated gene silencing of SKA1 on proliferation of hepatocellular carcinoma cells. Scand J Gastroenterol.

[25] Li J, Xuan JW, Khatamianfar V, Valiyeva F, Moussa M, Sadek A, Yang BB, Dong BJ, Huang YR, Gao WQ (2014). SKA1 over-expression promotes centriole over-duplication, centrosome amplification and prostate tumourigenesis. J Pathol.

[26] Sun W, Yao L, Jiang B, Guo L, Wang Q (2014). Spindle and kinetochore-associated protein 1 is overexpressed in gastric cancer and modulates cell growth. Mol Cell Biochem.

[27] Arai T, Okato A, Kojima S, Idichi T, Koshizuka K, Kurozumi A, Kato M, Yamazaki K, Ishida Y, Naya Y, Ichikawa T, Seki N (2017). Regulation of spindle and kinetochore-associated protein 1 by antitumor miR-10a-5p in renal cell carcinoma. Cancer Sci.

[28] Baumann C, Korner R, Hofmann K, Nigg EA (2007). PICH, a centromere-associated SNF2 family ATPase, is regulated by Plk1 and required for the spindle checkpoint. Cell.

[29] Zhang G, Yu Z, Fu S, Lv C, Dong Q, Fu C, Kong C, Zeng Y (2018). ERCC6L that is up-regulated in high grade of renal cell carcinoma enhances cell viability in vitro and promotes tumor growth in vivo potentially through modulating MAPK signalling pathway. Cancer Gene Ther.

[30] Scolz M, Widlund PO, Piazza S, Bublik DR, Reber S, Peche LY, Ciani Y, Hubner N, Isokane M, Monte M, Ellenberg J, Hyman AA, Schneider C, Bird AW (2012). GTSE1 is a microtubule plus-end tracking protein that regulates EB1-dependent cell migration. PLoS ONE.

[31] Bendre S, Rondelet A, Hall C, Schmidt N, Lin YC, Brouhard GJ, Bird AW (2016). GTSE1 tunes microtubule stability for chromosome alignment and segregation by inhibiting the microtubule depolymerase MCAK. J Cell Biol.

[32] Guo L, Zhang S, Zhang B, Chen W, Li X, Zhang W, Zhou C, Zhang J, Ren N, Ye Q (2016). Silencing GTSE-1 expression inhibits proliferation and invasion of hepatocellular carcinoma cells. Cell Biol Toxicol.

