# Supplementary figures and images for: Identification of biomarkers of chromophobe renal cell carcinoma by weighted gene co-expression network analysis
Source: Cancer Cell Int. 2018 Dec 17;18:206. doi: 10.1186/s12935-018-0703-z (PMC6296159; doi:10.1186/s12935-018-0703-z)

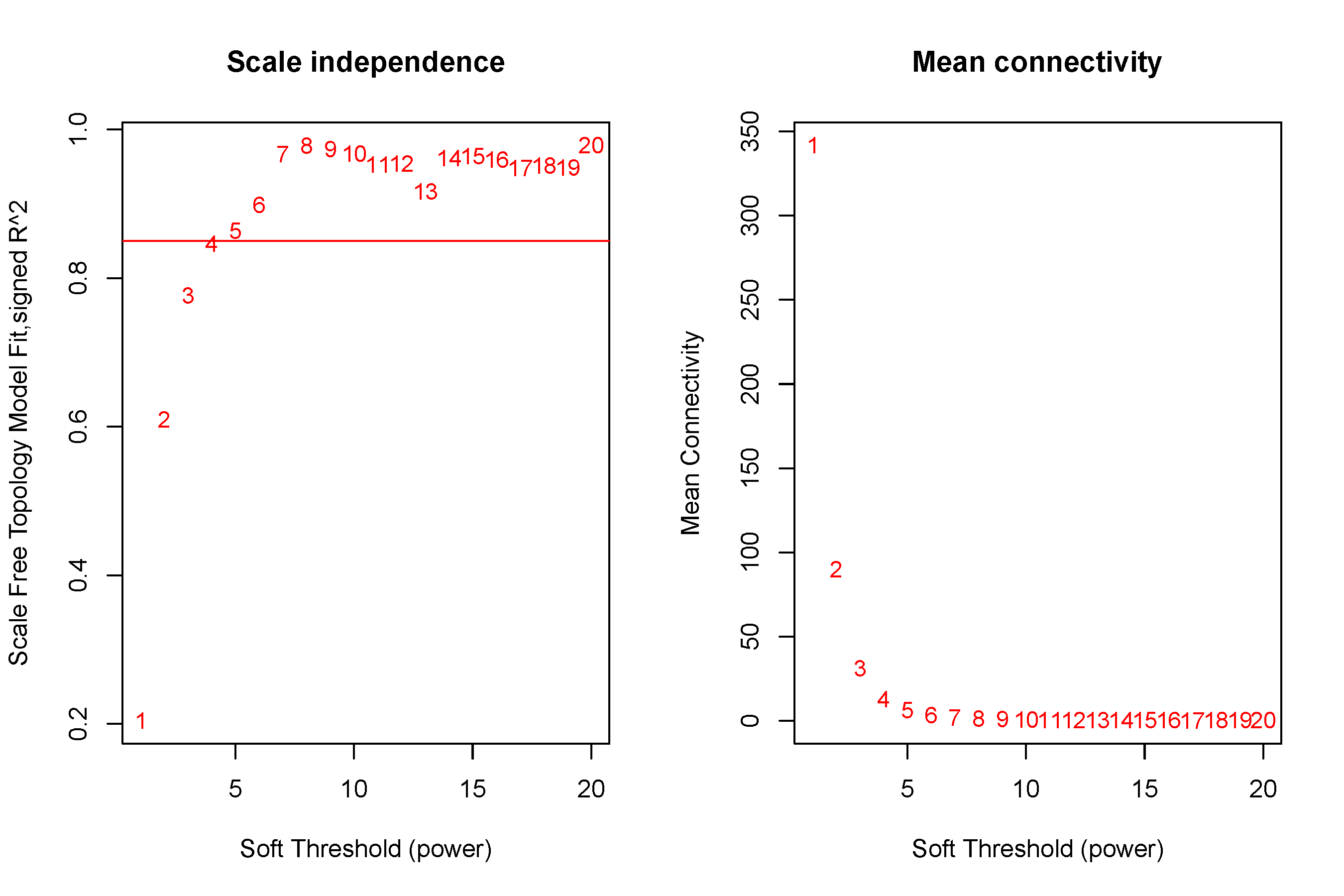

Supplement: Supplementary file 1 — Additional file 1: Fig S1. Analysis of network topology for various soft thresholding powers. The left panel shows the scale-free fit index (y-axis) as a function of the soft-thresholding power (x-axis). The right panel displays the mean connectivity (degree, y-axis) as a function of the soft-thresholding power (x-axis). [file 12935_2018_703_MOESM1_ESM.tif]

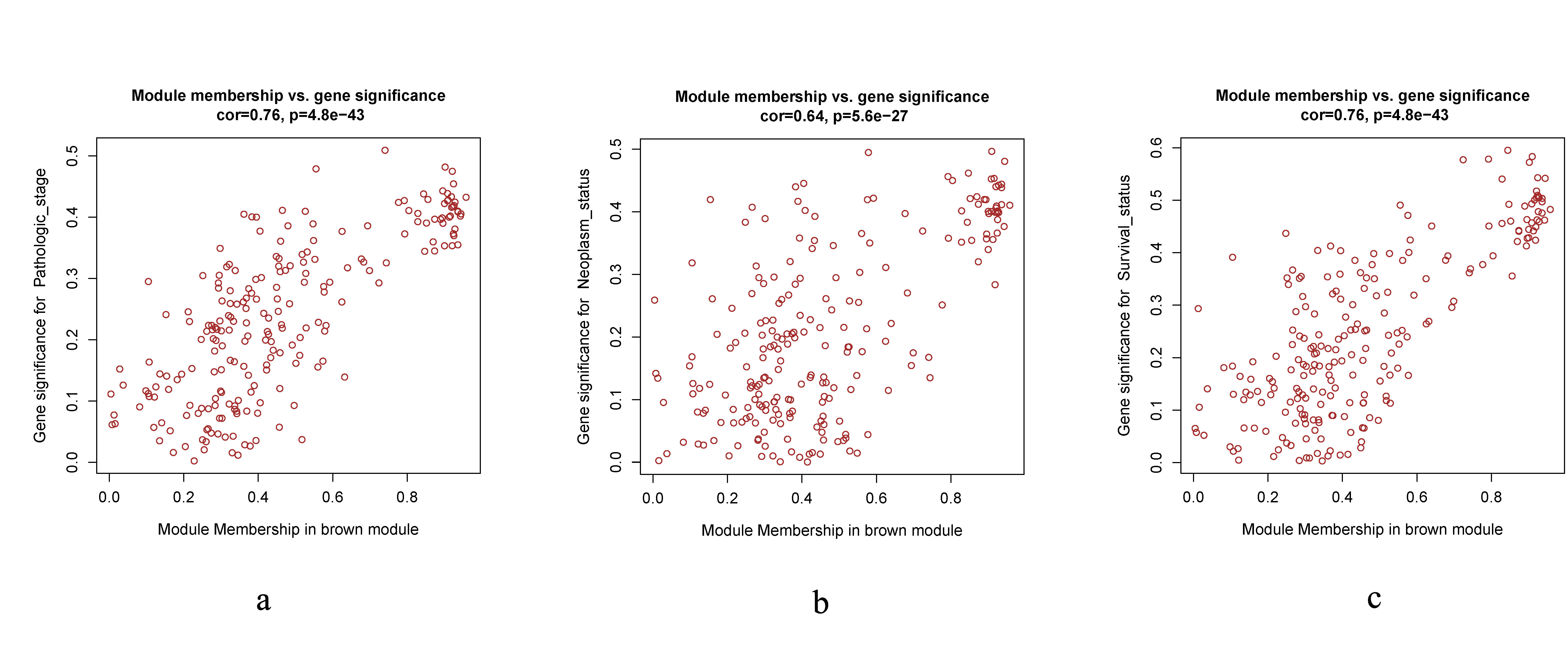

Supplement: Supplementary file 2 — Additional file 2: Fig S2. (a) Scatterplot of Gene Significance (GS) for pathologic stage vs. Module Membership (MM) in the brown module. (b) scatterplot of Gene Significance (GS) for neoplasm status vs. Module Membership (MM) in the brown module. (c) scatterplot of Gene Significance (GS) for survival status vs. Module Membership (MM) in the brown module. [file 12935_2018_703_MOESM2_ESM.tif]
