# Supplementary material for: Identification of biomarkers of chromophobe renal cell carcinoma by weighted gene co-expression network analysis
Source: Cancer Cell Int. 2018 Dec 17;18:206. doi: 10.1186/s12935-018-0703-z (PMC6296159; doi:10.1186/s12935-018-0703-z)
Supplement: Supplementary file 3 — Additional file 3: Table S1. GO enrichment analysis in brown module. [file 12935_2018_703_MOESM3_ESM.docx]

**Table S1. GO enrichment analysis in brown module**

| Term | Gene count | P-value | Genes in the test set |
| --- | --- | --- | --- |
| mitotic cell cycle phase transition | 12 | 7.43E-06 | CCNB2,CDT1,CDC45,MELK,CDKN2A,UBE2C,MCM10,  NEK2,CDC25C,FOXM1,PKMYT1,CDKN3 |
| mitotic spindle assembly | 6 | 1.28E-05 | TPX2,KIF4B,KIF4A,BIRC5,MYBL2,BIRC7 |
| mitotic spindle organization | 7 | 1.98E-05 | TPX2,KIF4B,KIF4A,BIRC5,MYBL2,TTK,BIRC7 |
| chromatin remodeling at centromere | 5 | 2.97E-05 | CENPV,CENPI,HJURP,CENPM,CENPA |
| centromere complex assembly | 5 | 5.26E-05 | CENPV,CENPI,HJURP,CENPM,CENPA |
| regulation of nuclear division | 4 | 9.24E-05 | NEK2,CDC25C,MKI67,PKMYT1 |
| mitotic nuclear division | 6 | 0.0002 | TPX2,KIF18B,UBE2C,BIRC5,MYBL2,BIRC7 |
| CENP-A containing nucleosome assembly | 4 | 0.0004 | CENPI,HJURP,CENPM,CENPA |
| CENP-A containing chromatin organization | 4 | 0.0004 | CENPI,HJURP,CENPM,CENPA |
| regulation of cell cycle process | 6 | 0.0005 | NEK2,KIF20A,CDC25C,FOXM1,MKI67,PKMYT1 |
