# Supplementary material for: Identification of biomarkers of chromophobe renal cell carcinoma by weighted gene co-expression network analysis
Source: Cancer Cell Int. 2018 Dec 17;18:206. doi: 10.1186/s12935-018-0703-z (PMC6296159; doi:10.1186/s12935-018-0703-z)
Supplement: Supplementary file 4 — Additional file 4: Table S2. KEGG pathways enrichment in brown module. [file 12935_2018_703_MOESM4_ESM.docx]

**Table S2. KEGG pathways enrichment in brown module**

| Term | Gene_count | P-value | Genes in the test set |
| --- | --- | --- | --- |
| Cell cycle | 10 | 1.26E-06 | CCNB2,CDC45,CDKN2A,E2F1,E2F2,TTK,CDC25C,PKMYT1,BUB1,SMC1B |
| Vitamin digestion and absorption | 3 | 0.0023 | CUBN,RBP2,APOA1 |
| Oocyte meiosis | 6 | 0.0025 | CCNB2,SGOL1,CDC25C,PKMYT1,BUB1,SMC1B |
| Glioma | 4 | 0.0059 | SHC4,CDKN2A,E2F1,E2F2 |
| Melanoma | 4 | 0.008 | CDKN2A,E2F1,E2F2,FGF21 |
| Chronic myeloid leukemia | 4 | 0.0089 | SHC4,CDKN2A,E2F1,E2F2 |
| Bladder cancer | 3 | 0.0105 | CDKN2A, E2F1,E2F2 |
| Complement and coagulation cascades | 4 | 0.0116 | F7,C5,SERPIND1,KNG1 |
| Mineral absorption | 3 | 0.019 | SLC9A3,MT1X,SLC26A9 |
| Progesterone-mediated oocyte maturation | 4 | 0.0237 | CCNB2,CDC25C,PKMYT1,BUB1 |
